# Supplementary material for: Differential Spatial Gene and Protein Expression Associated with Recurrence Following Chemoradiation for Localized Anal Squamous Cell Cancer
Source: Cancers (Basel). 2023 Mar 10;15(6):1701. doi: 10.3390/cancers15061701 (PMC10046657; doi:10.3390/cancers15061701)
Supplement: Supplementary file 1 [file cancers-15-01701-s001.zip › cancers-2210695-supplementary.pdf]

Supplementary Table 1: DSP protein panels

Human Immune Cell Typing	Immune cell typing of T cells, Regulatory T cells, B cells, Myeloid cells and NK cells.	CD20<br>GZMB<br>CD3<br>FOXP3<br>CD4	CD34<br>CD56<br>CD66b<br>CD8<br>Fibronectin
Immune Activation Status	Immune cell pro-inflammatory biomarkers and regulatory/inhibitory immune biomarkers	CD127<br>CD25<br>CD27<br>CD44<br>CD45RO	CD80<br>ICOS<br>PD-1<br>PD-L1<br>PD-L2
IO Drug Target	Immune oncology treatment targets	4-1BB<br>B7-H3<br>CTLA4<br>GITR<br>IDO1	LAG3<br>OX40L<br>STING<br>Tim-3<br>VISTA
MAPK Signaling	Cascades involved in cellular differentiation and inflammatory responses	Phospho-JNK (T183/Y185)<br>Phospho-MEK1 (S217/S221)<br>Phospho-p38 MAPK (T180/Y182)<br>BRAF<br>EGFR	Phospho-p44/42 MAPK ERK1/2 (T202/Y204)<br>Phospho-p90 RSK (T359/S363)<br>p44/42 MAPK ERK1/2<br>pan-RAS

PI3K/AKT Signaling	Signal transduction pathway associated to cell proliferation and survival	PLCG1<br>Phospho-GSK3A (S21)/Phospho-GSK3B (S9)<br>Phospho-Tuberin (T1462)<br>INPP4B<br>MET	Pan-AKT<br>Phospho-AKT1 (S473)<br>Phospho-GSK3B (S9)<br>Phospho-PRAS40 (T246)

Supplementary Table 2: DSP 84-plex RNA gene expression panel.

Gene	
4-1BB	GZMB
AKT1	HIF1A
ARG1	HLA-DQA1
B2M	HLA-DRB1
B7-H3	HLA-E
BATF3	ICAM1
BCL2	ICOSLG
CCL5	IDO1
CCND1	IFNAR1
CD3E	IFNG
CD4	IFNG
CD8A	IFNGR1
CD11b	IL6
CD11c	IL12B
CD20	IL15
CD27	ITGAV
CD40	ITGB2
CD40LG	ITGB8
CD44	Ki67
CD45	LAG3
CD47	LY6E
CD68	Multi-CK
CD74	PTEN
CD86	STAT1
CMKLR1	STAT2
CSF1R	STAT3

CTLA4	TBX21
CTNNB1	TIGIT
CXCL9	Tim3
CXCL10	TNF
CXCR6	VEGFA
DKK2	VISTA
EPCAM	Negative probes (1-8)
FAS	HK probes (OAZ1, POLR2A, RAB7A,SDHA, UBB)
FOXP3	
